# Editorial: Neuroscience and the media

**DOI:** 10.3389/fnins.2023.1327123

**Published:** 2023-11-17

**Authors:** Celia Andreu-Sánchez, Miguel Ángel Martín-Pascual, José María Delgado-García

**Affiliations:** ^1^Neuro-Com Research Group, Department of Audiovisual Communication and Advertising, Universitat Autònoma de Barcelona, Barcelona, Spain; ^2^Research and Development, Institute of Spanish Public Television (RTVE), Corporación Radio Televisión Española, Barcelona, Spain; ^3^Division of Neurosciences, University Pablo de Olavide, Seville, Spain

**Keywords:** neurocinematics, neuroscience, visual perception, media content, neurocommunication

We watch media content constantly, and yet little is known about how the brain manages the perception of this type of content. Since the 1950s, when Gastaut et al. (1952); Cohen-Séat et al. (1954) and Gastaut and Bert (1954) started to analyze the brain activity of subjects viewing movies with different narratives, much interdisciplinary work has been done. Learning how media affect spectators can have an impact not only in the neurocinematics field of study (Hasson et al., 2008), but also in the understanding of human communication (Murch, 1995; Nakano et al., 2009; Andreu-Sánchez et al., 2018). With this Research Topic, we had the goal of increasing the knowledge about how the brain perceives and processes media content. We thought that this could be of great interest both for media creators who would benefit from a knowledge of perceptual patterns, and for scientists using media content as stimuli in their investigations, who would have more information when designing their stimuli and the variables in them; it would also be of great interest for clinical purposes, since learning the correlations of audio-visual content and brain behavior could inspire new insights.

The different manuscripts included within this topic show several interesting results.

One paper proposes finding physiological markers of viewers' perception and correlating them to short film ratings. The authors reveal some EEG (such as the positive correlation of beta/alpha with film ratings) and peripheral markers (such as facial muscles) that reflect viewers' rating and can predict them to a certain extent (Kosonogov et al.). Another work addresses neural correlates of continuity editing, and finds that the scale of the cuts in the editing affects brain activity. The authors find that edits with an increased scale lead to amplification of the event-related potential (ERP) deflection, while scale reduction leads to decreases, compared with edits keeping the scale across cuts (Sanz-Aznar et al.). In a related work, the impact that camera movements have on audiences is studied. On this regard, results are mixed: while movement made by cameras affects the viewers' sense of involvement, those same movements do not necessarily increase emotional responses in viewers (Yilmaz et al.).

The sound is also investigated to compare perception in viewers when listening to monophonic, stereo, and surround modes. The surround presentation mode shows higher event-related desynchronization (ERD) in alpha and low-beta in the centro-parietal area. The authors suggest that this may be related with an embodied simulation mechanism (Langiulli et al.). Another work proves that the presence of an unfamiliar person while listening to audios modulates the perception: a more homogeneous perception pattern is found when listening to audios when alone, and a more heterogeneous behavior is shown when the listening is done with another person present (Kauttonen et al.).

Advertising communication is also approached in this Research Topic. One work studies differential neural reward reactivity in response to food advertising in children. The authors carry out an experimental proposal using fMRI with children aged 9–12 years old with food and non-food dynamic and static ads, and find significantly higher responses in certain areas, such as the right and left hemispheres of the amygdala and insula for the dynamic food ad medium (Yeum et al. a, b). The authors conclude that the advertising medium has specific effects on neural response to food cues. Another study examines attitudes toward political advertising. Through three experiments, the authors look at differences in social vigilantism and the need for cognition. They found that higher levels of social vigilantism would be related to greater intentions to counterargue and better memory for attitude-incongruent information (Miller et al.).

Additionally, there is an investigation aimed at studying the functional effects of steady-state visual evoked potentials (SSVEP) elicited by rhythmic visual stimulation (RVS) on visuospatial selective attention, since those have been used as biomarkers in studies of neural processing based on the assumption that they would not affect cognition. Here the authors find that target discriminatory accuracy and reaction time vary significantly across the RVS frequency (Li et al.). Another study uses eye-tracking data to modify training of deep convolutional neural networks, and thus change the models' visual attention during object recognition in natural images. The authors present a novel approach to visual perception that can have an impact in neuroscience and in computer science studies (van Dyck et al.).

The topic also includes a paper that investigates the role of spontaneous theory of mind on the processing of dramatic irony scenes in films. Its authors suggest that exposure to undisclosed critical information in cinema enhances the frequency of spontaneous epistemic state inferences and integration into event models of exploitation (Cabañas et al.). Another work provides an overview of approaches to the study of the media while it introduces an organizing scheme that connects the causal path from media content to brain responses and to media effects. In that manuscript, the authors argue the need for creating a new substantive science at the intersection of media and neuroscience (Schmälzle and Huskey).

Finally, media professionalization is studied in a work that finds beta-band differences in primary motor cortex between media and non-media professionals when watching motor actions in movies, suggesting that media expertise could be related with beta-band activity in motor actions (Andreu-Sánchez et al.). This could be of interest in brain-computer interface training contexts.

Overall, this Research Topic includes several works aimed at learning more about how media can be studied from a neuroscientific perspective.

## Author contributions

CA-S: Conceptualization, Project administration, Writing—original draft, Writing—review & editing. MM-P: Conceptualization, Project administration, Writing—original draft, Writing—review & editing. JD-G: Conceptualization, Project administration, Writing—original draft, Writing—review & editing.
